# Modeling vaccination strategies with limited early COVID-19 vaccine access in low- and middle-income countries: A case study of Thailand

**DOI:** 10.1016/j.idm.2023.11.003

**Published:** 2023-11-15

**Authors:** Suparinthon Anupong, Tanakorn Chantanasaro, Chaiwat Wilasang, Natcha C. Jitsuk, Chayanin Sararat, Kan Sornbundit, Busara Pattanasiri, Dhammika Leshan Wannigama, Mohan Amarasiri, Sudarat Chadsuthi, Charin Modchang

**Affiliations:** a Biophysics Group, Department of Physics, Faculty of Science, Mahidol University, Bangkok, 10400, Thailand; b Center for Disease Modeling, Faculty of Science, Mahidol University, Bangkok, 10400, Thailand; c Ratchaburi Learning Park, King Mongkut’s University of Technology Thonburi, Ratchaburi, Thailand; d Department of Physics, Faculty of Liberal Arts and Science, Kasetsart University Kamphaeng Saen Campus, Nakhon Pathom, 73140, Thailand; e Department of Infectious Diseases and Infection Control, Yamagata Prefectural Central Hospital, Yamagata, Japan; f Department of Microbiology, Faculty of Medicine, Chulalongkorn University, King Chulalongkorn Memorial Hospital, Thai Red Cross Society, Bangkok, Thailand; g Center of Excellence in Antimicrobial Resistance and Stewardship, Faculty of Medicine, Chulalongkorn University, Bangkok, Thailand; h School of Medicine, Faculty of Health and Medical Sciences, The University of Western Australia, Nedlands, Western Australia, Australia; i Biofilms and Antimicrobial Resistance Consortium of ODA Receiving Countries, The University of Sheffield, Sheffield, United Kingdom; j Pathogen Hunter’s Research Collaborative Team, Department of Infectious Diseases and Infection Control, Yamagata Prefectural Central Hospital, Yamagata, Japan; k Laboratory of Environmental Hygiene, Department of Health Science, School of Allied Health Sciences/Graduate School of Medical Sciences, Kitasato University, Kitasato, Sagamihara-Minami, Kanagawa, 252-0373, Japan; l Department of Physics, Faculty of Science, Naresuan University, Phitsanulok, 65000, Thailand; m Centre of Excellence in Mathematics, Ministry of Higher Education, Science, Research and Innovation, Bangkok, 10400, Thailand; n Thailand Center of Excellence in Physics, Ministry of Higher Education, Science, Research and Innovation, 328 Si Ayutthaya Road, Bangkok, 10400, Thailand

**Keywords:** COVID-19, Low- and middle-income countries, Vaccine inequality, Thailand

## Abstract

Low- and middle-income countries faced significant challenges in accessing COVID-19 vaccines during the early stages of the pandemic. In this study, we utilized an age-structured modeling approach to examine the implications of various vaccination strategies, vaccine prioritization, and vaccine rollout speeds in Thailand, an upper-middle-income country experiencing vaccine shortages during the early stages of the pandemic. The model directly compares the effectiveness of several vaccination strategies, including the heterologous vaccination where CoronaVac (CV) vaccine was administered as the first dose, followed by ChAdOx1 nCoV-19 (AZ) vaccine as the second dose, under varying disease transmission dynamics. We found that the traditional AZ homologous vaccination was more effective than the CV homologous vaccination, regardless of disease transmission dynamics. However, combining CV and AZ vaccines via either parallel homologous or heterologous vaccinations was more effective than relying solely on AZ homologous vaccination. Additionally, prioritizing vaccination for the elderly aged 60 years and above was the most effective way to reduce mortality when community transmission is well-controlled. On the other hand, prioritizing workers aged 20–59 was most effective in lowering COVID-19 cases, irrespective of the transmission dynamics. Lastly, despite the vaccine prioritization strategy, rapid vaccine rollout speeds were crucial in reducing COVID-19 infections and deaths. These findings suggested that in low- and middle-income countries where early access to high-efficacy vaccines might be limited, obtaining any accessible vaccines as early as possible and using them in parallel with other higher-efficacy vaccines might be a better strategy than waiting for and relying solely on higher-efficacy vaccines.

## 1 Introduction

The pandemic of the coronavirus disease 2019 (COVID-19) has left a profound imprint on global health, economies, and societies. Although the World Health Organization (WHO) officially lifted the designation of COVID-19 as a public health emergency of international concern (PHEIC) on May 5, 2023 (Zarocostas, 2023), it is crucial to acknowledge that the global threat posed by COVID-19 persists. The virus has transitioned into an endemic state and remains an ongoing health challenge in many nations (Biancolella et al., 2023; Looi, 2023; Wong, Zoller, Fouda, & Paolucci, 2023). While vaccination and non-pharmaceutical interventions stand as the most formidable barrier against the spread of the disease, its success largely depends on equitable access and appropriate administration (Andrews et al., 2022; Gardner & Kilpatrick, 2021; Higdon et al., 2021; Liu, Qin, Liu, & Liu, 2021; Lopez Bernal et al., 2021; Sheikh, McMenamin, Taylor, & Robertson, 2021).

The first year of COVID-19 vaccine availability historically saw a stark global disparity. While around 58% of the world’s population received at least one vaccine dose by the end of 2021, over 70% of individuals in wealthy nations had received the primary vaccination regimen (Hannah Ritchie, 2020; Moore, Hill, Dyson, Tildesley, & Keeling, 2022). In sharp contrast, a mere 4% of individuals in low-income countries received at least a single vaccine dose. Furthermore, a distinct difference was observed in the types of vaccines administered in high-income nations, where mRNA vaccines predominated due to their heightened efficacy, while low- and middle-income countries primarily relied on inactivated virus vaccines (Maxmen, 2021; Tregoning, Flight, Higham, Wang, & Pierce, 2021).

As an upper-middle-income country, Thailand experienced these challenges firsthand, especially with vaccine shortages in the early phases of its vaccination drive. When the national vaccination campaign started in June 2021, Thailand faced difficulties ensuring an adequate vaccine supply (Chookajorn, Kochakarn, Wilasang, Kotanan, & Modchang, 2021; Hannah Ritchie, 2020). Notably, during this period, the vaccination campaign in Thailand relied predominantly on the administration of the CoronaVac vaccine developed by Sinovac Biotech Ltd. (CV) and the ChAdOx1 nCoV-19 vaccine (Vaxzevria) manufactured by AstraZeneca plc. (AZ). However, the emergence of the Delta variant, coupled with the limited availability of the AZ vaccine, prompted the Department of Disease Control of Thailand to implement a series of vaccination strategies to address the ongoing transmission concerns (Chookajorn et al., 2021). One such strategy involved adopting a heterologous vaccination approach, where the CV vaccine was administered as the first dose, followed by a second dose of the AZ vaccine (Organization, 2022). Although the CV + AZ heterologous vaccination was found to induce higher levels of anti-RBD IgG antibodies than that of two-dose homologous CV + CV vaccines (Wanlapakorn, Suntronwong, Phowatthanasathian, Yorsaeng, Thongmee, et al., 2022) and could help accelerate the vaccination speed as the second dose could be administered 3 weeks after the first dose compared to 12 weeks in the traditional AZ homologous vaccination, there is still no study to assess the effectiveness of this CV + AZ heterologous vaccination over implementing the traditional separate CV and AZ homologous vaccination regimens.

In this study, we used Thailand as a case study to delve into the potential vaccination strategies to mitigate the disparities in early vaccine accessibility in low- and middle-income nations. Specifically, we evaluated various vaccination strategies, emphasizing the heterologous CV + AZ approach Thailand adopted during the early periods of its vaccination program. To achieve these goals, we first delineated the course and timeline of COVID-19 transmission and vaccination in Thailand. An age-structured compartmental model for COVID-19 transmission was then employed to assess the effectiveness of different vaccination strategies. Finally, the impact of vaccine prioritization strategies was also investigated.

## 2 Materials and methods

### 2.1 Data sources

We collected data on COVID-19 cases, deaths, and vaccination from publicly available sources. Specifically, we retrieved the numbers of confirmed COVID-19 cases and deaths in Thailand from the Department of Disease Control, Ministry of Public Health, Thailand (OurWorldinData, 2022b; The Department of Disease Control, 2022). Additionally, data on vaccine administration, including the numbers of the first, second, and booster doses, were obtained from public data repositories (OurWorldinData, 2022a; Vantanaprason, 2022). We also collected information on administered vaccine doses by the manufacturers, sourced from reference (Vantanaprason, 2022).

### 2.2 COVID-19 transmission and vaccination model

**Fig. 1:**
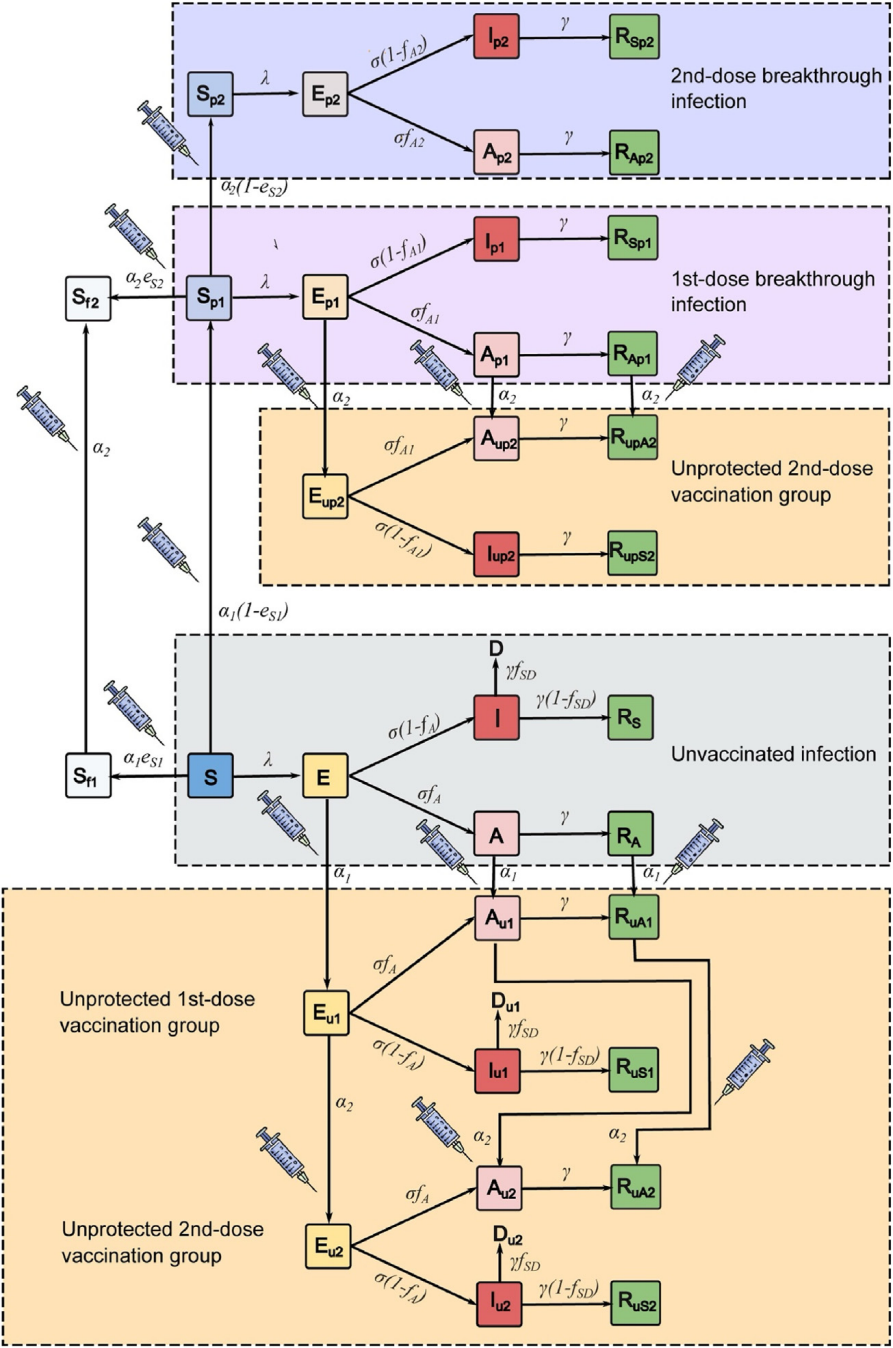
Illustration of the COVID-19 transmission model with two-dose vaccination. The diagram depicts a COVID-19 transmission model involving two doses of vaccination. The model consists of six main epidemiological compartments: susceptible (S), exposed (E), asymptomatic infectious (A), symptomatic infectious (I), asymptomatic recovered (R_A_), and symptomatic recovered (R_S_). Within each main epidemiological compartment, subscripts 1 and 2 indicate one-dose and two-dose vaccination, respectively. Subscripts u, p, and f denote the extent of protection conferred by vaccination: unprotected, partially protected, and fully protected, respectively. Individuals who were initially exposed, asymptomatic infectious, or asymptomatic recovered and later received vaccines are assumed to have acquired protection through prior infection rather than vaccination. Therefore, they are categorized into the unprotected vaccination groups, shown in yellow areas. Transmission among unvaccinated individuals is depicted in the gray area. The pink and purple areas indicate breakthrough infections after the first and second doses of vaccination, respectively. Detailed model equations for each colored area can be found as equations S1-S4 in Supplementary Information A, and a summary of model parameters is provided in Table S1.

We developed an age-structure compartmental model with two-dose vaccination to analyze the dynamics of COVID-19 transmission in Thailand. The schematic of the model is shown in Fig. 1. The model encompasses six primary epidemiological compartments: susceptible (S), exposed (E), asymptomatic infectious (A), symptomatic infectious (I), asymptomatic recovered (R_A_), and symptomatic recovered (R_S_). Based on the available contact data in Thailand (Prem et al., 2017), the population in each compartment was classified into 16 age groups with a five-year interval (0–4, 5–9, …, 70–74, and ≥75 years). All individuals were assumed to be initially susceptible to SARS-CoV-2 infection. When susceptible individuals are infected, with the force of infection λ, they immediately transition to the exposed compartment. The exposed individuals then move to the infectious compartment with a rate *σ* that is inversely proportional to the latent period. The infectious individuals can be symptomatic or asymptomatic. The proportion of asymptomatic infections (*f*_*A*_) was assumed to be the same for all age groups. A fraction of *f*_*SD*_ of symptomatic infectious individuals dies with a rate of *γ* that is inversely proportional to the infectious period. The age-specific *f*_*SD*_ was estimated from the age-specific infection fatality rate (*IFR*) obtained from references (Bubar et al., 2021; Levin et al., 2020).

We assumed that only individuals in the S, E, A, and R_A_ compartments could be vaccinated; however, vaccine-induced immunity only plays a role in vaccinated susceptible individuals. Vaccinated individuals in the E, A, and R_A_ compartments will be protected by infection-induced immunity (Gazit et al., 2022). After susceptible individuals are vaccinated, they move to either a fully (S_f_) or partially (S_p_) infection-protected susceptible compartment with the ratio of S_f_ and S_p_ determined by the efficacy against infection of the vaccine (*e*_*S*_). The parameters $\alpha_{1}$ and $\alpha_{2}$ are the rollout speeds per capita for the first and the second dose of vaccines, respectively.

The force of infection, $\lambda_{i}$, for individuals in age group *i* is given by

(1) $$\lambda_{i}=\sum_{j=1}^{n}\beta_{ij}\left(\frac{I_{j}+q_{A}A_{j}+\left(1-e_{I1}\right)I_{p1j}+q_{A}\left(1-e_{I1}\right)A_{p1j}+\left(1-e_{I2}\right)I_{p2j}+q_{A}\left(1-e_{I2}\right)A_{p2j}}{N_{j}-D_{j}-D_{u1j}-D_{u2j}}\right)\text{,}$$

where $\beta_{ij}$ is the transmission rate from infectious individuals in age group *i* to susceptible individuals in age group *j*, which is proportional to the contact frequency ($c_{ij}$) (Diekmann & Heesterbeek, 2000; Prem et al., 2017). *q*_*A*_ represents the relative infectiousness of asymptomatic individuals compared to symptomatic ones. The vaccine efficacies against infection, transmission, and symptomatic disease from the first vaccination dose are denoted as $e_{S1},e_{I1}\text{,}$ and $e_{D1}$, respectively. $e_{S2},e_{I2}\text{,}$ and $e_{D2}$ are the additional vaccine efficacies induced from the second vaccination dose. The terms (*1 – e*_*I1*_) and (*1 – e*_*I2*_) represent the transmission contributed from the infectious individuals who have already been vaccinated for one and two doses, respectively. *N*_*j*_ is the total number of individuals in age group *j*, and *D*_*j*_ is the number of individuals in age group *j* who have died. Subscripts 1 and 2 denote one-dose and two-dose vaccination, respectively, while subscripts u, p, and f represent the varying degrees of vaccination protection: unprotected, partially protected, and fully protected, respectively (Fig. 1). The model equations describing the vaccination and transmission dynamics are detailed in Supplementary Information A. The simulations were performed and analyzed using MATLAB R2020b. All model parameters are summarized in Table S1.

### 2.3 Homologous and heterologous vaccination strategies

We considered three vaccination strategies for homologous vaccination, namely, homologous CV vaccination (CV + CV), homologous AZ vaccination (AZ + AZ), and parallel homologous CV and homologous AZ vaccination (CV and AZ parallel). The vaccines can be distributed to two target groups, namely, workers (20–60 years) and elders (≥60 years), with different prioritization strategies. The vaccination starts at the beginning of the simulation (*t* = 0). According to the vaccination roadmap announced by the Thai government in July 2021 (Panarat Thepgumpanat, 2021b), the vaccine rollout speed was set at a constant speed of 10 million doses/month. The recommended time intervals between the first and second doses of the vaccines ($d_{dose}$) were set to 21 and 84 days for the CV vaccine (Organization, 2021; April) and the AZ vaccine (Voysey et al., 2021), respectively.

For the heterologous vaccination strategy, since the first available COVID-19 vaccine in Thailand was the CV vaccine, we, therefore, investigated the scenario where the CV vaccine is used as the first dose, followed by the AZ vaccine as the second dose with the dosing interval ($d_{dose}$) of 21 days (Wanlapakorn, Suntronwong, Phowatthanasathian, Yorsaeng, Vichaiwattana, et al., 2022). As the individuals who got the CV + AZ heterologous vaccination have a similar level of immunity as the homologous AZ + AZ vaccination (Wanlapakorn, Suntronwong, Phowatthanasathian, Yorsaeng, Vichaiwattana, et al., 2022), we assumed that the vaccine efficacies of the first dose and second dose of heterologous vaccination correspond to the efficacies of one dose of CV and two doses of AZ vaccine, respectively. In these heterologous vaccination scenarios, the disease transmission was assumed to start when the AZ vaccine was available.

To accelerate vaccination, all vaccine available initially was assumed to be delivered as the first dose without reserving for the second dose during the first day of vaccination (*t* = 0) to the last day of the time interval (*t* = $d_{dose}$) (Mak & TinglongTang, 2021). After that, all vaccine supplies would be administered as the second dose. The vaccination would return to the first dose when no more individuals were due for the second dose. The alternating vaccination of the first and second doses was repeated until the desired vaccination coverage was achieved. The vaccine rollout speed for the first ($\alpha_{1}$) and second ($\alpha_{2}$) doses for each time step was calculated from the ratio of the rollout speed and the number of individuals to be vaccinated (see Supplementary Information B.).

### 2.4 Prioritization strategies

To investigate the vaccine prioritization strategies in Thailand, we considered three different prioritization strategies: (1) no prioritization, where all individuals over 20 years old are randomly vaccinated; (2) elder prioritization, where individuals aged 60 years and older receive vaccination first, followed by those aged 20 to 59; and (3) worker prioritization, where individuals aged 20 to 59 are vaccinated first, followed by those aged 60 years and older. This section of the study focuses on homologous AZ vaccination (AZ + AZ) using the model illustrated in Fig. 1, and we evaluated the impact of various rollout speeds, including 50,000, 100,000, 250,000, and 500,000 doses per day.

### 2.5 Estimating the percentages of infections and deaths averted by vaccination

We calculated the reduction in deaths and cases using the following formulas:

$$\text{Reduction}\;\text{in}\;\text{deaths}\left(\%\right)=\left(\frac{D_{n}-D_{v}}{D_{n}}\right)\times 100\%\text{,}$$

$$\text{Reduction}\;\text{in}\;\text{cases}\left(\%\right)=\left(\frac{C_{n}-C_{v}}{C_{n}}\right)\times 100\%\text{.}$$

Here, *D*_*v*_ and *D*_*n*_ are mean cumulative deaths simulated with and without vaccination, while *C*_*v*_, and *C*_*n*_ are mean cumulative cases simulated with and without vaccination, respectively. Furthermore, a sensitivity analysis was conducted to assess the variations in reductions of both deaths and cases. For this analysis, simulations were executed utilizing the 95% lower and upper bounds of vaccine efficacies (Table S1).

### 2.6 Estimation of the time-varying reproduction number

We used the “EpiEstim” package in R software (Version 4.2.2) and applied a statistical method developed by Cori et al. to estimate the time-varying reproduction number, *R*_*t*_ (Cori et al., 2020; Gostic et al., 2020). To estimate the *R*_*t*_, we only required the daily count of new confirmed cases and the serial interval distribution, which we assumed to be a discretized Gamma distribution with an average of 3.96 days and a standard deviation of 4.75 days (Nishiura, Linton, & Akhmetzhanov, 2020).

## 3 Results

### 3.1 Vaccination and COVID-19 situations in Thailand

**Fig. 2:**
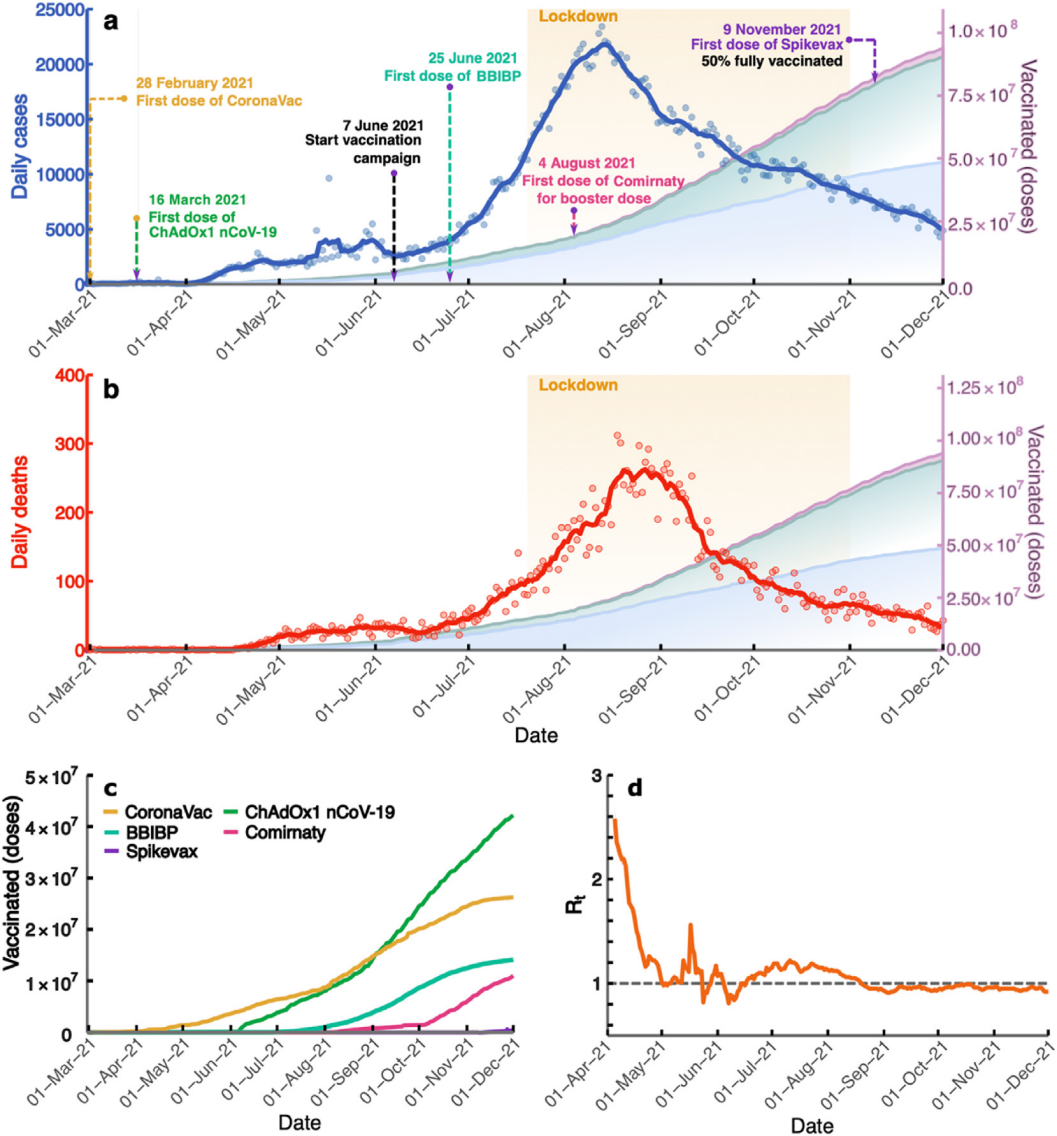
Vaccination and COVID-19 situations in Thailand. The blue line (a) and the red line (b) illustrate the number of daily new cases and the number of daily deaths, respectively. The blue, green, and purple areas show the cumulative numbers of first, second, and booster doses. The black-dashed arrows indicate the time point when the vaccination campaign started. The colored dashed arrows point to the start date of administering the vaccines from the different manufacturers. The yellow highlight area shows the duration of the lockdown measure. (c) Cumulative doses of vaccines from different manufacturers that have been delivered. (d) The time-varying reproduction numbers starting from April 1 to December 1, 2021. The line shows the median of *R*_*t*_, and the shaded area indicates the 95% CI. The horizontal dashed line indicates the *R*_*t*_ threshold value of 1.

Since the first case of COVID-19 was detected in Thailand in early 2020, the country has experienced two well-managed and controlled waves of the epidemic (Chookajorn et al., 2021; Wilasang et al., 2020; Wilasang, Jitsuk, et al., 2022), resulting in a cumulative total of 29,127 cases and 95 deaths as of March 29, 2021. However, in early April 2021, the third transmission wave began, caused by the imported Alpha variant, followed by the fourth wave in late May 2021, caused by the Delta variant (Tribune, 2021; Kunno et al., 2021; Ndeh, Tesfaldet, Budnard, & Chuaicharoen, 2022; Rarueysong, 2021). These two imported variants caused the largest outbreak of COVID-19 in Thailand since the pandemic began (Wilasang, Modchang, Lincharoen, & Chadsuthi, 2022), with a peak of 21,838 confirmed cases on August 7, 2021 (Fig. 2a), and a peak of 312 confirmed deaths on August 18, 2021 (Fig. 2b). To control the spread of the virus, the government implemented lockdown measures from July 20 to November 1, 2021 (Thailand, 2020; Wannigama, Amarasiri, Hongsing, et al., 2023) (yellow highlight in Fig. 2a and b).

Regarding the COVID-19 vaccination in Thailand, the first doses of CV and AZ vaccines were delivered to the healthcare workers on February 28 and March 16, respectively, while the national vaccination campaign started on June 7. Later, on July 25, the Sinopharm BBIBP vaccines were imported by the Chulabhorn Royal Academy for sale and charity (Reuters, 2021a). The Comirnaty from Pfizer-BioNTech COVID-19 vaccine, donated by the United States, was used as booster doses for healthcare workers who got the two doses of CV vaccine and children aged 12–18 years. Additionally, Spikevax from Moderna, imported by a private hospital in Thailand, was administered on November 9, when the transmission wave had already gone down. Due to the shortage of vaccines at the beginning of the vaccination campaign in Thailand, many strategies of the heterologous vaccination were employed; the CV vaccine was used as the first dose and the AZ vaccine as the second dose. At the end of 2021, around 44 million, 26.4 million, and 17.1 million doses of AZ, CV, and Comirnaty were administered, respectively (Fig. 2c). The numbers of cumulative first (blue), second (green), and booster (purple) doses are shown in Fig. 2a and b.

We estimated the time-varying reproduction number (*R*_*t*_), the average number of secondary cases an infected person generates. A value of *R*_*t*_ greater than the threshold value of 1 indicates that new infections are growing at time *t*, whereas *R*_*t*_ less than 1 indicates that the epidemic size is shrinking at time *t*. We employed a method proposed by Cori and colleagues (Cori, Ferguson, Fraser, & Cauchemez, 2013; Wilasang, Jitsuk, et al., 2022) to estimate the *R*_*t*_ in Thailand. *R*_*t*_ was calculated from April 5, when daily cases exceeded 100, to December 1, 2021 (Fig. 2d). We found that the median *R*_*t*_ in the early transmission phase was 2.57 (95% CI: 2.36–2.80). However, Thailand implemented lockdown measures in early April 2021, resulting in the R_*t*_ decreasing to a value below one during that time. In July 2021, the number of deaths and cases drastically increased due to the spread of the more transmissible Delta variant. As a result, the *R*_*t*_ value rose to a value greater than one. The Thai government then expanded the lockdown areas to other parts of the country and increased the vaccination speed (Reuters, 2021b). After Thailand launched the national COVID-19 vaccination program, the *R*_*t*_ decreased to a value below one on August 26, 2021, and has since remained below one until December 1, 2021.

### 3.2 Impacts of homologous and heterologous vaccination strategies on mortality and infections

**Fig. 3:**
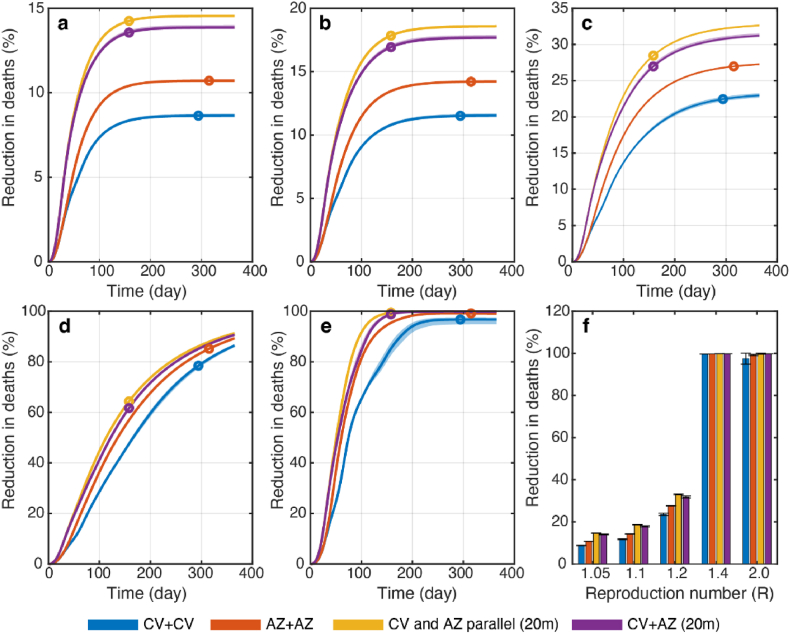
Reduction in cumulative deaths under different vaccination strategies. Results were obtained from model simulations with the reproduction number of (a) *R* = 1.05, (b) 1.1, (c) 1.2, (d) 1.4, and (e) 2.0 with either CV + CV (blue), AZ + AZ (red) homologous vaccinations, parallel homologous vaccination of CV and AZ (yellow), and CV + AZ heterologous vaccination (purple). The times at which 70% of individuals were vaccinated are indicated by colored dots. (f) The comparison of the reduction in deaths at the equilibrium under different vaccination scenarios. Error bars and shaded areas represent the range of variation in cumulative deaths due to uncertainties in vaccine efficacies. This variation was obtained through sensitivity analysis, which involved simulations using the 95% lower and upper bounds of vaccine efficacies.

**Fig. 4:**
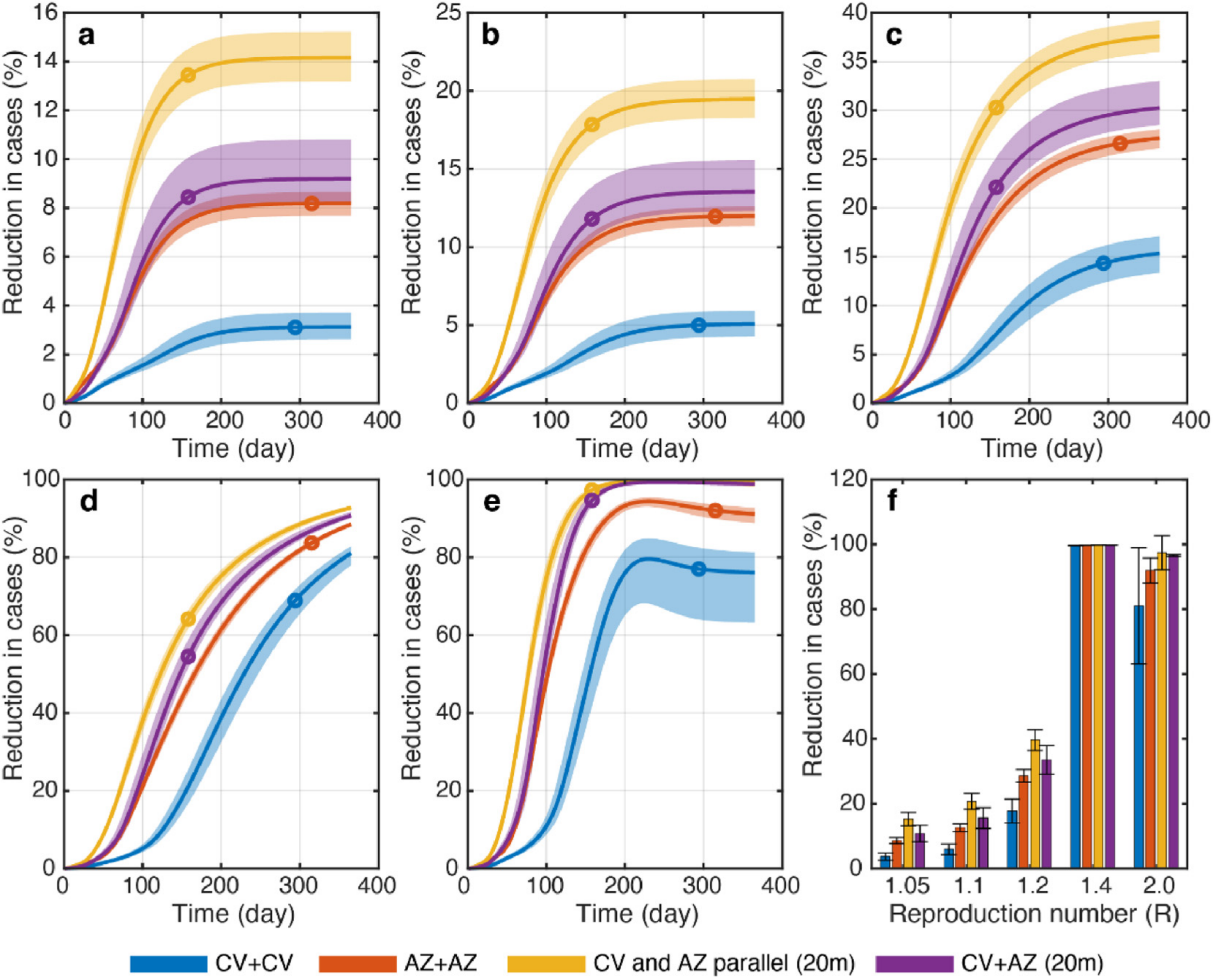
Reduction in cumulative cases under different vaccination strategies. The cumulative numbers of cases were from model simulations with the reproduction number of (a) *R* = 1.05, (b) 1.1, (c) 1.2, (d) 1.4, and (e) 2.0 with either CV + CV (blue), AZ + AZ (red) homologous vaccinations, parallel homologous vaccination of CV and AZ (yellow), and CV + AZ heterologous vaccination (purple). Color dots indicate the times at which 70% of individuals were vaccinated. (f) The comparison of the reduction in cumulative cases at the equilibrium under different vaccination scenarios. Error bars and shaded areas indicate the range of variation in cumulative cases due to uncertainties in vaccine efficacies. This variation was determined through sensitivity analysis, which entailed simulations incorporating the 95% lower and upper bounds of vaccine efficacies.

We examined the effect of homologous and heterologous vaccination strategies on reducing cumulative deaths and cases. Three different scenarios were explored for the homologous vaccination: i) homologous CV (CV + CV), ii) homologous AZ (AZ + AZ), and iii) parallel homologous CV and homologous AZ vaccinations (CV and AZ parallel). For the heterologous vaccination, we explored the strategy in which the population gets the CV vaccine as the first dose and the AZ vaccine as the second dose. Fig. 3 shows the reduction in the cumulative number of deaths due to different vaccination strategies. For the homologous vaccination strategies, our results indicated that the AZ + AZ strategy could advert more deaths than the CV + CV strategy across the entire range of reproduction numbers (Fig. 3f). However, in the scenarios where the CV and AZ vaccines were employed, the parallel CV and AZ homologous vaccination performed slightly better than the CV + AZ heterologous vaccination, especially when the effective reproduction number was below 1.4 (Fig. 3f). Similar results were also found regarding reducing the number of cumulative cases (Fig. 4). Note, however, that since both CV and AZ vaccines were concurrently employed in CV + AZ heterologous and parallel CV and AZ homologous vaccination strategies, the vaccination rate for these two strategies was, therefore, two times higher than that of the homologous CV and homologous AZ vaccination strategies.

### 3.3 Impact of vaccine prioritization strategies and vaccine rollout speeds

We conducted an analysis of three distinct strategies for prioritizing vaccine distribution: (1) No prioritization, where vaccines are randomly administered to all individuals over the age of 20; (2) Elder prioritization, prioritizing individuals aged 60 years and older, followed by those aged 20 to 59; and (3) Worker prioritization, which first vaccinates individuals aged 20 to 59, followed by those aged 60 years and older. We assessed the impact of these prioritization strategies by examining their effect on reducing the cumulative number of deaths and cases at equilibrium. The distribution of vaccines was carried out at varying rates, including 50,000, 100,000, 250,000, and 500,000 doses per day, reflecting a range of vaccination capacities in Thailand.

**Fig. 5:**
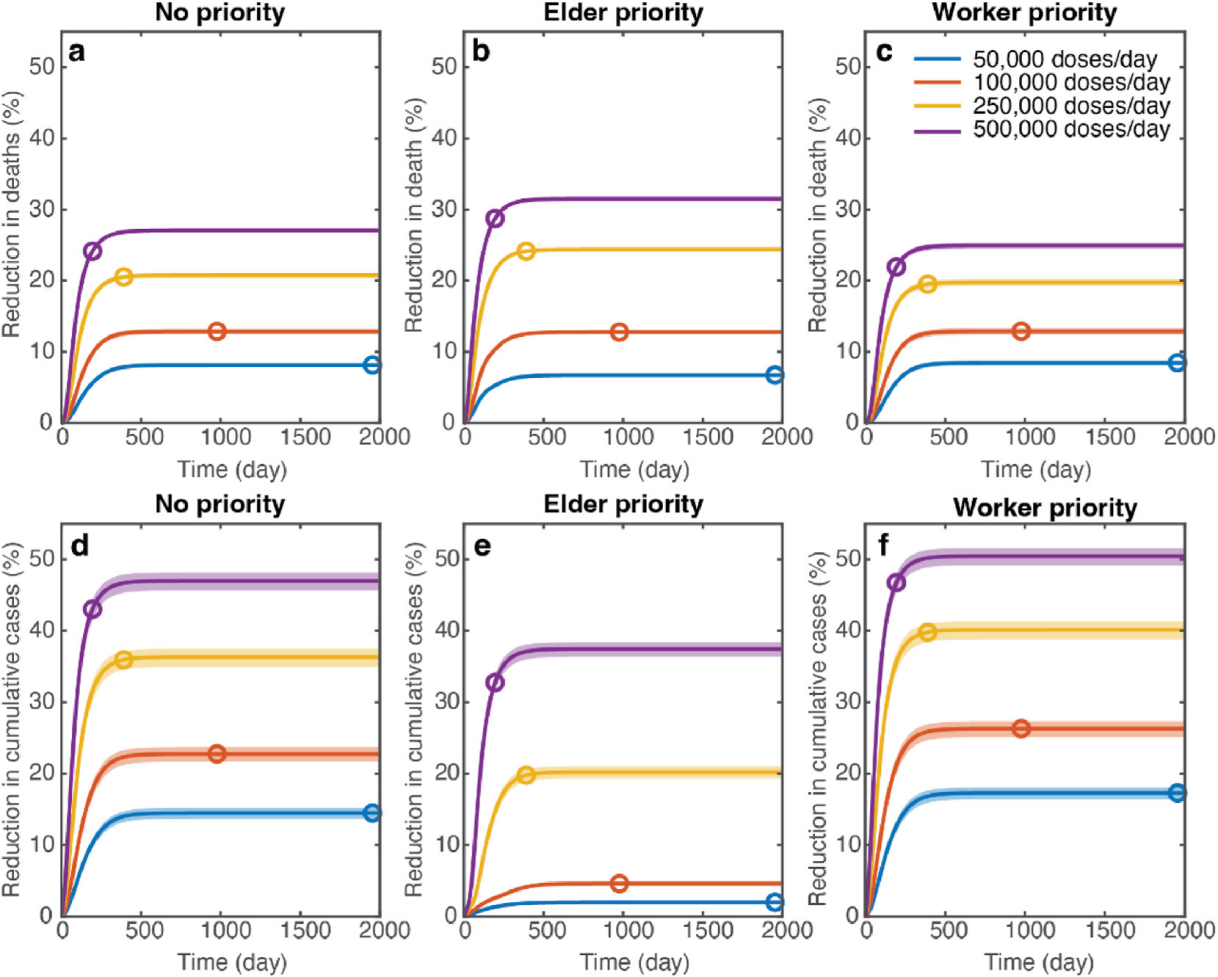
Impact of vaccine prioritization strategies on mortality and infections. (a–c) Reductions in cumulative deaths and (d–f) reductions in cumulative cases simulated using *R* = 1.2 under different vaccine rollout speeds. (a, d), (b, e), and (c, f) show the results under the random vaccine distribution (no priority), elderly priority, and worker priority, respectively. The color dots on the graph indicate the times when 70% of the total population was fully vaccinated. Shaded areas represent the variation in cumulative cases and deaths due to uncertainties in vaccine efficacies. This variation was derived through sensitivity analysis, which involved simulations using the 95% lower and upper bounds of vaccine efficacies.

Fig. 5 shows the impacts of different prioritization strategies on mortality and infections. Our analysis showed that a faster vaccine rollout speed resulted in a greater reduction in mortality and infection for all prioritization strategies. In the high-supply scenario with a rollout speed of 500,000 doses per day, the elder prioritization strategy was the most effective in reducing deaths compared to other strategies. Conversely, in the low-supply scenario with a rollout speed of 50,000 doses per day, prioritizing vaccines for the elderly resulted in the smallest effect in reducing infections and deaths (Fig. 5b and e). In this scenario, the reduction in cumulative cases was maximized when vaccines were first allocated to the working age group.

**Fig. 6:**
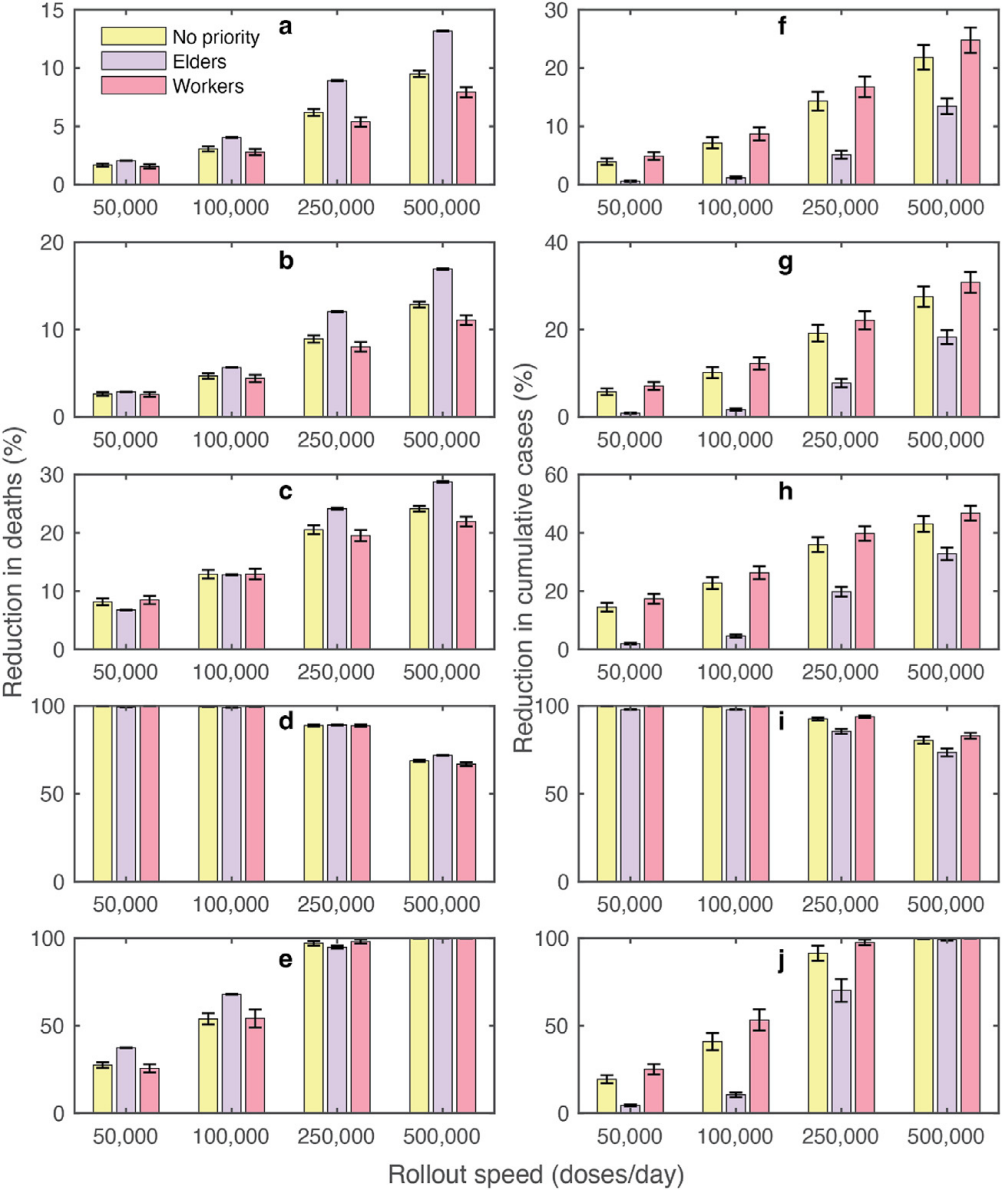
Effects of vaccine prioritization strategies on COVID-19 mortality and infections. (a–e) Percentage reduction in cumulative deaths for varying vaccine rollout speeds in the scenario where *R* = 1.05, 1.1, 1.2, 1.4, and 2.0, respectively. (f–j) Percentage reduction in cumulative cases for varying vaccine rollout speeds in the scenario where *R* = 1.05, 1.1, 1.2, 1.4, and 2.0, respectively. Error bars indicate the range of variation in cumulative cases and deaths due to uncertainties in vaccine efficacies.

To investigate the impact of the vaccine prioritization strategies on mortality and infections under different disease transmission dynamics, we varied the reproduction numbers and measured the percentage reductions in cumulative deaths and cases (Fig. 6). Our analysis showed that for slow transmission dynamics (*R* = 1.05, 1.1, and 1.2), prioritizing the elderly aged 60 years and above resulted in a greater reduction in COVID-19 mortality than other strategies for all vaccine rollout speeds (Fig. 6a–c). When disease transmission was at *R* = 1.4, all vaccine prioritization strategies led to similar reductions in mortality (Fig. 6d). In this case, the reductions in deaths for the elder and worker prioritization strategies were 97% and 98%, respectively. For very fast transmission dynamics (*R* = 2.0), vaccinating the elderly first was more effective in preventing deaths when the vaccine rollout speed was low (<100,000 doses/day) (Fig. 6e). However, in a high-supply scenario with a rollout speed of 250,000–500,000 doses/day, all vaccine prioritization strategies were similar in reducing mortality. In contrast, the results revealed that the highest reduction in the case numbers can always be achieved by prioritizing the vaccination of working-age individuals (Fig. 6f–j).

## 4 Discussion

This research outlines Thailand’s first nine months of vaccination, which serves as a case study for the challenges low- and middle-income countries faced in accessing COVID-19 vaccines during the pandemic. During the first year of COVID-19 vaccination, less than 10% of the population in low-income countries had received at least one vaccine dose, while most people in wealthier nations were fully vaccinated, highlighting the issue of vaccine inequity (Chookajorn et al., 2021; Mathieu et al., 2021; Moore et al., 2022) (Fig. S1). Thailand, an upper-middle-income country, also encountered a vaccine shortage during the initial stages of its national vaccination campaign, which began on June 7, 2021 (OurWorldinData, 2022c), almost six months after the World Health Organization (WHO) granted emergency use authorization for the first COVID-19 vaccine.

Thailand implemented various vaccination strategies to combat SARS-CoV-2 transmission with limited vaccine availability, including a heterologous vaccination strategy where CV was the first dose and AZ was the second (Panarat Thepgumpanat, 2021a). To evaluate the effectiveness of the vaccination strategies employed during the initial stages of the national vaccination campaign in Thailand, we developed an age-structured model that directly compares the effectiveness of several vaccination strategies under varying disease transmission dynamics. Our findings indicated that, when measuring the cumulative numbers of cases and deaths averted by vaccination, the AZ + AZ strategy was more effective than the CV + CV strategy for the traditional homologous vaccinations, regardless of the reproduction number (Fig. 3f). This is likely due to the higher efficacy of both the first and second doses of the AZ vaccine. However, combining CV and AZ vaccines via either parallel homologous or heterologous vaccinations was more effective than relying solely on the AZ vaccine. Additionally, faster vaccine rollout speed led to a greater reduction in both deaths and cases (Fig. 5). These findings suggested that in low- and middle-income countries where early access to high-efficacy vaccines might be limited, obtaining affordable vaccines as early as possible and using them in parallel with other vaccines might be a better strategy for preventing severe outcomes.

We discovered that shortening the dosing interval for the CV + AZ heterologous vaccination to three weeks, compared to twelve weeks for AZ homologous vaccination, could speed up fully vaccinating individuals. However, this vaccination strategy did not result in a higher reduction in cumulative deaths. In contrast, using parallel homologous vaccinations of CV and AZ was slightly more effective in reducing cumulative deaths, especially when non-pharmaceutical interventions were implemented to lower the effective reproduction number to less than 1.4. This could be attributed to the high first-dose efficacy of the AZ vaccine, which renders reducing the inter-dose duration via heterologous vaccination less advantageous. These findings are consistent with other studies examining the optimal duration between the first and second doses of COVID-19 vaccines, which suggested that a second dose can be delayed by ≥8 weeks for vaccines with first-dose efficacy greater than 50% (Liu et al., 2022; Silva, Sagastizábal, Nonato, Struchiner, & Pereira, 2021). Additionally, a vaccination strategy that distributes a single vaccine dose to more people could be more effective at averting deaths than a traditional two-dose vaccination campaign if the first vaccine dose is highly efficacious and introduced under stringent social distancing interventions (Matrajt et al., 2021).

We also investigated the impact of the vaccine prioritization strategies on COVID-19 mortality and infections. Our results suggested that a faster vaccine rollout speed could rapidly reduce more COVID-19 infections and deaths in all vaccine prioritization strategies. Thus, the fast rollout speed of the vaccine is critical to quickly curbing the spread of COVID-19. Moreover, we discovered that the most effective vaccine prioritization strategy depends on the reproduction number of the COVID-19 transmission. For the slow transmission dynamics (maybe due to intense non-pharmaceutical interventions), prioritizing the elderly can reduce COVID-19 mortality more than other strategies for all vaccine rollout speeds, consistent with previous research (Bubar et al., 2021; Matrajt et al., 2021). In the scenario where the transmission dynamics are fast (*R* = 2.0) and the rollout speed of the vaccine is fast (in the range of 250,000–500,000 doses/day), all vaccine prioritization strategies were equally effective at reducing mortality. However, if the vaccine rollout speed is slow (in the range of 50,000–100,000 doses/day), vaccinating the elderly first can drastically reduce mortality more than other strategies (Fig. 6), which aligns with previous work (Galli et al., 2023). Indeed, Thailand has implemented a very strict lockdown combined with case isolation and non-pharmaceutical interventions such as physical distancing and face masking during the COVID-19 outbreak (Chookajorn et al., 2021; Wilasang et al., 2020; Wilasang, Jitsuk, et al., 2022). This has resulted in a low effective reproduction number during the outbreak (Fig. 2d). As a result, vaccination strategies targeting the elderly in Thailand could be optimal for reducing COVID-19 mortality.

In terms of vaccine prioritization to reduce COVID-19 infections, our results suggested that prioritizing vaccines among workers (aged 20–59 years) could lower the number of COVID-19 cases more than the other strategies. This aligns with previous studies that also recommend prioritizing essential workers due to their increased risk of exposure (Buckner, Chowell, & Springborn, 2021; Chen et al., 2021; Minoza, Bongolan, & Rayo, 2021; Shim, 2021). Our results demonstrate that prioritizing worker vaccinations results in a reduction in COVID-19 cases, regardless of the reproduction number considered (Fig. 6f–j).

There are several limitations in our study that need to be acknowledged. Firstly, our model did not explicitly account for the emergence of new variants of the virus, which could potentially affect vaccine effectiveness and transmission dynamics. The emergence of new variants could alter the outcomes of different vaccination strategies and necessitate the adaptation of vaccination policies in response to the evolving situation (Liu et al., 2022; Wannigama, Amarasiri, Phattharapornjaroen, et al., 2023). Secondly, our model assumed constant vaccine effectiveness over time, ignoring the possibility of waning immunity, which may lead to an underestimation of the disease burden after COVID-19 vaccination is implemented. In addition, waning immunity could affect the long-term effectiveness of different vaccination strategies and the required frequency of booster doses (Sararat, Wangkanai, Wilasang, Chantanasaro, & Modchang, 2022). Thirdly, we focused on Thailand as a case study, and our results may not be generalizable to other low- and middle-income countries with different demographic and epidemiological profiles. Factors such as population age structure, healthcare infrastructure, and the prevalence of underlying health conditions can affect the optimal vaccination strategy for a given country (Bubar et al., 2021; Matrajt et al., 2021). Finally, our analysis did not consider potential changes in individual behavior and the evolving use of non-pharmaceutical interventions during the outbreak. These factors could influence transmission dynamics and the rollout of vaccination efforts. However, since our study did not aim to reconstruct the complete COVID-19 transmission dynamics in Thailand, incorporating these elements would have unnecessarily complicated the model. Despite these limitations, our research provides valuable insights for public health officials and policymakers in low- and middle-income countries dealing with initial vaccine shortages. The findings underscore the importance of tailoring vaccination strategies to the local context, considering vaccine availability, deployment pace, and transmission dynamics.

## 5 Conclusion

This study investigated the impact of homologous and heterologous vaccination strategies, vaccine prioritization, and rollout speeds on COVID-19 infections and mortality in Thailand. This upper-middle-income country faced challenges in accessing vaccines during the early stages of the pandemic. Our findings indicated that employing parallel homologous or heterologous vaccination strategies with all available CV and AZ vaccines was more effective in controlling COVID-19 than relying solely on AZ vaccination. Moreover, faster vaccine rollout speeds were crucial in reducing COVID-19 infections and deaths across all vaccine prioritization strategies. The effectiveness of vaccine prioritization strategies depended on the reproduction number of COVID-19 transmission. Prioritizing the elderly population for vaccination was the most effective strategy for reducing mortality if the ongoing transmission in the community was well-controlled with stringent non-pharmaceutical interventions. In contrast, prioritizing workers (aged 20–59 years) was the most effective strategy for reducing the number of COVID-19 cases, regardless of the reproduction number considered.

## Data availability

The data used in this study are available in the online Supplementary files.

## CRediT authorship contribution statement

**Suparinthon Anupong:** Data curation, Formal analysis, Investigation, Methodology, Software, Visualization, Writing – original draft. **Tanakorn Chantanasaro:** Data curation, Resources, Validation, Writing – original draft. **Chaiwat Wilasang:** Data curation, Resources, Validation, Writing – original draft. **Natcha C. Jitsuk:** Data curation, Resources, Validation, Writing – original draft. **Chayanin Sararat:** Data curation, Resources, Validation, Writing – original draft. **Kan Sornbundit:** Validation, Writing – original draft. **Busara Pattanasiri:** Validation, Writing – original draft. **Dhammika Leshan Wannigama:** Validation, Writing – review & editing. **Mohan Amarasiri:** Validation, Writing – review & editing. **Sudarat Chadsuthi:** Conceptualization, Data curation, Formal analysis, Methodology, Software, Visualization, Writing – original draft, Writing – review & editing. **Charin Modchang:** Conceptualization, Funding acquisition, Methodology, Project administration, Supervision, Writing – original draft, Writing – review & editing.

## Declaration of competing interest

The authors declare that they have no competing interests.
