# Supplementary material for: Modeling vaccination strategies with limited early COVID-19 vaccine access in low- and middle-income countries: A case study of Thailand
Source: Infect Dis Model. 2023 Nov 15;8(4):1177–89. doi: 10.1016/j.idm.2023.11.003 (PMC10709621; doi:10.1016/j.idm.2023.11.003)
Supplement: Multimedia component 1 [file mmc1.pdf]

Supplementary Information:

Modeling vaccination strategies with limited early COVID-19 vaccine access in low- and middle-income countries: A case study of Thailand

Suparinthon Anupong[1,2], Tanakorn Chantanasaro[1,2], Chaiwat Wilasang[1,2], Natcha C. Jitsuk[1,2], Chayanin Sararat[1,2], Kan Sornbundit[2,3], Busara Pattanasiri[2,4], Dhammika Leshan Wannigama[5,6,7,8,9,10], Mohan Amarasiri[11], Sudarat Chadsuthi[2,12*], and Charin Modchang[1,2,13,14*]

[1]Biophysics Group, Department of Physics, Faculty of Science, Mahidol University, Bangkok 10400, Thailand

[2] Center for Disease Modeling, Faculty of Science, Mahidol University, Bangkok, 10400, Thailand

[3]Ratchaburi Learning Park, King Mongkut's University of Technology Thonburi, Ratchaburi, Thailand

[4]Department of Physics, Faculty of Liberal Arts and Science, Kasetsart University Kamphaeng Saen Campus, Nakhon Pathom 73140, Thailand

[5]Department of Infectious Diseases and Infection Control, Yamagata Prefectural Central Hospital, Yamagata, Japan

[6]Department of Microbiology, Faculty of Medicine, Chulalongkorn University, King Chulalongkorn Memorial Hospital, Thai Red Cross Society, Bangkok, Thailand

[7]Center of Excellence in Antimicrobial Resistance and Stewardship, Faculty of Medicine, Chulalongkorn University, Bangkok, Thailand

[8]School of Medicine, Faculty of Health and Medical Sciences, The University of Western Australia, Nedlands, Western Australia, Australia

[9]Biofilms and Antimicrobial Resistance Consortium of ODA receiving countries, The University of Sheffield, Sheffield, United Kingdom

[10]Pathogen Hunter's Research Collaborative Team, Department of Infectious Diseases and Infection Control, Yamagata Prefectural Central Hospital, Yamagata, Japan

[11]Laboratory of Environmental Hygiene, Department of Health Science, School of Allied Health Sciences/Graduate School of Medical Sciences, Kitasato University, Kitasato, Sagamihara-Minami, Kanagawa, 252-0373, Japan

[12]Department of Physics, Faculty of Science, Naresuan University, Phitsanulok 65000, Thailand.

[13]Centre of Excellence in Mathematics, Ministry of Higher Education, Science, Research and Innovation, Bangkok 10400, Thailand

[14]Thailand Center of Excellence in Physics, Ministry of Higher Education, Science, Research and Innovation, 328 Si Ayutthaya Road, Bangkok 10400, Thailand

* To whom correspondence should be addressed.

E-mail: charin.mod@mahidol.edu (C.M.); sudaratc@nu.ac.th (S.C.)

A. Model equations

Our transmission model comprises six primary epidemiological compartments: susceptible (S), exposed (E), asymptomatic infectious (A), symptomatic infectious (I), asymptomatic recovered (R_A), and symptomatic recovered (R_S). We categorized the population within each compartment into 16 age groups, each spanning five-year intervals, except for the last age group, which includes individuals aged ≥ 75 years (0-4, 5-9, …, 70-74, and ≥ 75 years). Additionally, we stratified the population according to their infection and vaccination statuses, as illustrated in **Figure 1**. The model can be described by the following set of differential equations, grouped into four categories that correspond to different infection and transmission dynamics, corresponding to distinct colored regions in **Figure 1**:

1. **Unvaccinated infection**: COVID-19 transmission among individuals who have not been vaccinated is described by the following equations:

$$\left.\begin{aligned}
\dot{S}_i &= -\lambda_i S_i - \alpha_{1i} S_i \\
\dot{E}_i &= \lambda_i S_i - \sigma E_i - \alpha_{1i} E_i \\
\dot{A}_i &= \sigma f_A E_i - \gamma A_i - \alpha_{1i} A_i \\
\dot{I}_i &= \sigma (1 - f_A) E_i - \gamma I_i \\
\dot{D}_i &= f_{SD} \gamma I_i \\
\dot{R}_{A,i} &= \gamma A_i - \alpha_{1i} R_{A,i} \\
\dot{R}_{S,i} &= (1 - f_{SD}) \gamma I_i
\end{aligned}\right\} \qquad \text{(S1)}$$

2. **1st-dose breakthrough infection**: COVID-19 transmission among individuals who have received the 1st- dose of a vaccine is described by the following equations:

$$\left.\begin{aligned}
\dot{S}_{p1,i} &= \alpha_{1i}(1 - e_{S1}) S_i - \lambda_i S_{p1,i} - \alpha_{2i} e_{S2} S_{p1,i} \\
\dot{S}_{f1,i} &= \alpha_{1i} e_{S1} S_i \\
\dot{E}_{p1,i} &= \lambda_i S_{p1,i} - \sigma E_{p1,i} - \alpha_{2i} E_{p1,i} \\
\dot{A}_{p1,i} &= \sigma f_{A1} E_{p1,i} - \gamma A_{p1,i} - \alpha_{2i} A_{p1,i} \\
\dot{I}_{p1,i} &= \sigma (1 - f_{A1}) E_{p1,i} - \gamma I_{p1,i} \\
\dot{R}_{Ap1,i} &= \gamma A_{p1,i} - \alpha_{2i} R_{Ap1,i} \\
\dot{R}_{Sp1,i} &= \gamma I_{p1,i}
\end{aligned}\right\} \qquad \text{(S2)}$$

3. **2nd-dose breakthrough infection**: COVID-19 transmission among individuals who have received the 2nd-dose of vaccine is described by the following equations:

$$\left.\begin{aligned}
&\dot{S}_{p2,i} = \alpha_{2i}(1-e_{S2})S_{p1,i} - \lambda_i S_{p2,i}\\
&\dot{S}_{f2,i} = \alpha_{2i}e_{S2}S_{p1,i}\\
&\dot{E}_{p2,i} = \lambda_i S_{p2,i} - \sigma E_{p2,i}\\
&\dot{A}_{p2,i} = \sigma f_{A2}E_{p2,i} - \gamma A_{p2,i}\\
&\dot{I}_{p2,i} = \sigma(1-f_{A2})E_{p2,i} - \gamma I_{p2,i}\\
&\dot{R}_{Ap2,i} = \gamma A_{p2,i}\\
&\dot{R}_{Sp2,i} = \gamma I_{p2,i}
\end{aligned}\right\} \qquad \text{(S3)}$$

4. **Unprotected vaccination**: Individuals who were initially exposed, asymptomatic infectious, or asymptomatic recovered and later received vaccines are assumed to have acquired protection through prior infection rather than vaccination. COVID-19 transmission among individuals in this group is described by the following equations:

$$\left.\begin{aligned}
&\dot{E}_{u1,i} = \alpha_{1i}E_i - \sigma E_{u1,i} - \alpha_{2i}E_{u1,i}\\
&\dot{A}_{u1,i} = \alpha_{1i}A_i + \sigma f_A E_{u1,i} - \gamma A_{u1,i} - \alpha_{2i}A_i\\
&\dot{I}_{u1,i} = \sigma(1-f_A)E_{u1,i} - \gamma I_{u1,i}\\
&\dot{D}_{u1,i} = f_{SD}\gamma I_{u1,i}\\
&\dot{R}_{Au1,i} = \alpha_{1i}R_{A,i} + \gamma A_{u1,i} - \alpha_{2i}R_{Au1,i}\\
&\dot{R}_{Su1,i} = (1-f_{SD})\gamma I_{u1,i}\\
&\\
&\dot{E}_{u2,i} = \alpha_{2i}E_{u1,i} - \sigma E_{u2,i}\\
&\dot{A}_{u2,i} = \alpha_{2i}A_i + \sigma f_A E_{u2,i} - \gamma A_{u2,i}\\
&\dot{I}_{u2,i} = \sigma(1-f_A)E_{u2,i} - \gamma I_{u2,i}\\
&\dot{D}_{u2,i} = f_{SD}\gamma I_{u2,i}\\
&\dot{R}_{Au2,i} = \alpha_{2i}R_{Au1,i} + \gamma A_{u2,i}\\
&\dot{R}_{Su2,i} = (1-f_{SD})\gamma I_{u2,i}\\
&\\
&\dot{E}_{up2,i} = \alpha_{2i}E_{p1,i} - \sigma E_{up1,i}\\
&\dot{A}_{up2,i} = \alpha_{2i}A_{p1,i} + \sigma f_{A1}E_{up1,i} - \gamma A_{up1,i}\\
&\dot{I}_{up2,i} = \sigma(1-f_{A1})E_{up1,i} - \gamma I_{up1,i}\\
&\dot{R}_{Aup2,i} = \alpha_{2i}R_{Ap1,i} + \gamma A_{up1,i}\\
&\dot{R}_{Sup2,i} = \gamma I_{up1,i}
\end{aligned}\right\} \qquad \text{(S4)}$$

Subscripts 1 and 2 serve to distinguish between single-dose and two-dose vaccinations, while subscripts u, p, and f denote the degree of protection offered by vaccination, representing unprotected, partially protected, and fully protected states, respectively (refer to **Figure 1** for visual reference). The index i, ranging from 1 to 16, designates different age groups, i.e., i = 1 represents individuals aged 0 to 4 years old, while i = 16 represents the population aged 75 years and older.

B. Model parameters

To examine the impact of homologous and heterologous vaccination strategies in Thailand, it is crucial to consider the vaccine efficacies of CoronaVac (CV) and ChAdOx1-nCoV-19 (AZ). These vaccine efficacies are key factors in understanding the role of COVID-19 vaccines in mitigating the outbreak, encompassing their effectiveness in preventing infection, symptomatic disease, and onward transmission (Lipsitch & Kahn, 2021; Ssentongo et al., 2022).

In our study, we categorized vaccine efficacies into three main parameters: efficacy against symptomatic disease (e_D), infection (e_S), and transmission (e_I), both for the first and second vaccine doses (e_{S1}, e_{I1}, e_{D1}, and e_{S2}, e_{I2}, e_{D2} in **Table S1**). Unfortunately, there is limited literature on clinical trials evaluating the efficacy of the first and second doses of CV and AZ vaccines specifically in Thailand. Therefore, we utilized vaccine efficacy data from studies conducted in Chile and the UK for CV and AZ vaccines, respectively (Eyre et al., 2022; Jara et al., 2021; Lopez Bernal et al., 2021; Pritchard et al., 2021). Notably, these vaccine efficacies have demonstrated a consistent trend across different countries. For instance, the efficacies of CV observed in Chile align with those reported in Brazil and Indonesia (Fadlyana et al., 2021; Palacios et al., 2021). Similarly, studies conducted in São Paulo have reported AZ efficacies within the same range as those observed in the UK (Hitchings et al., 2021). As such, we chose to adopt these vaccine efficacies as the basis for our research in Thailand. The summarized values of these vaccine efficacies, along with all other model parameters, are provided in **Table S1**.

Table S1. Parameters and their default values used in the model.

Parameter	Definition	Value	Reference(s)
$1/\sigma$	Latent period	3 days	(Davies et al., 2020)
$1/\gamma$	Symptomatic and asymptomatic infectious period	5 days	(Davies et al., 2020)
q_A	Relative asymptomatic infectiousness	0.5	(Byambasuren et al., 2020)
f_A	Fraction of infected individuals who become asymptomatic	0.5	(Byambasuren et al., 2020)
f_{A1}	Fraction of one-dose-vaccinated infected individuals who become asymptomatic	See equation (S6)	
f_{A2}	Fraction of two-dose-vaccinated infected individuals who become asymptomatic	See equation (S7)	
IFR	Infection fatality ratio	See equation (S9)	(Levin et al., 2020)
f_{SD}	Proportion of symptomatic infected individuals who eventually die	See equation (S8)	(Bubar et al., 2021)
e_S	Two-dose vaccine efficacy against infection	0.80 (95% CI, 0.74 – 0.84) for AZ,<br>0.659 (0.652 – 0.666) for CV	(Eyre et al., 2021; Lopez Bernal et al., 2021),

Parameter	Definition	Value	Reference(s)
			(Eyre et al., 2021; Jara et al., 2021)
e_I	Two-dose vaccine efficacy against transmission	0.58 (0.52 – 0.62) for AZ,<br>0.513 (0.139 – 0.710) for CV	(Eyre et al., 2022)<br>Estimated
e_D	Two-dose vaccine efficacy against symptomatic disease	0.67 (0.613 – 0.718) for AZ,<br>0.59 (0.16 – 0.816) for CV	(Lopez Bernal et al., 2021),<br>(Eyre et al., 2021; Jara et al., 2021)
e_{S1}	One-dose vaccine efficacy against infection	0.56 (0.51 – 0.61) for AZ,<br>0.155 (0.142 – 0.168) for CV	(Eyre et al., 2021; Lopez Bernal et al., 2021),<br>(Eyre et al., 2021; Jara et al., 2021)
e_{I1}	One-dose vaccine efficacy against transmission	0.31 (0.28 – 0.34) for AZ,<br>0.138 (–0.602 – 0.548) for CV	(Eyre et al., 2022)<br>Estimated
e_{D1}	One-dose vaccine efficacy against symptomatic disease	0.30 (0.243 – 0.353) for AZ,	(Lopez Bernal et al., 2021),

Parameter	Definition	Value	Reference(s)
		0.138 (–0.602 – 0.548) for CV	(Eyre et al., 2021; Jara et al., 2021)
e_{S2}	Second-dose incremental vaccine efficacy against infection	$1-(1-e_S)/(1-e_{S1})$	
e_{I2}	Second-dose incremental vaccine efficacy against transmission	$1-(1-e_I)/(1-e_{I1})$	
e_{D2}	Second-dose incremental vaccine efficacy against symptomatic disease	$1-(1-e_D)/(1-e_{D1})$	
R	Reproduction number	1.05, 1.10, 1.20, 1.40, 1.80, 2.00	
α_1	First vaccine dose rollout speed	See equation (S10-S11)	
α_2	Second vaccine dose rollout speed	See equation (S12-S13)	
d_{dose}	Time interval between the first and second doses of vaccines	84 days (AZ),<br>21 days (CV)	(Moghadas et al., 2021; Voysey et al., 2021),<br>(Organization, 2021, Arpil)

As there is no available data regarding the efficacy of the CV vaccine against transmission, we adopted the assumption that e_{I1} is equivalent to e_{D1}. We estimated e_I by utilizing the e_I/e_D ratio from the AZ vaccine. It's important to note that for the lower bounds of CV vaccine efficacies, both e_{D1} and e_{I1} had negative values. To prevent introducing negative vaccine efficacy estimates into the model, which could arise from potential biases in efficacy estimation, we imposed constraints to ensure that the values of e_{D1} and e_{I1} remained at or above zero throughout our simulations.

The vaccine efficacy against disease is estimated as a reduction in symptomatic disease of infected individuals in the vaccinated population compared to those in the unvaccinated population, as follows (Lipsitch & Kahn, 2021; Ssentongo et al., 2022)

$$e_{D1} = \frac{I-I_{p1}}{I}, \tag{S5}$$

where I and I_{p1} are the number of symptomatic infected individuals in the unvaccinated and 1st-dose vaccinated groups, respectively, and can be written as

$$e_{D1} = \frac{\sigma(1-f_A)-((1-e_{S1})\sigma(1-f_{A1}))}{\sigma(1-f_A)},$$

therefore

$$f_{A1} = 1 - \frac{(1-e_{D1})(1-f_A)}{(1-e_{S1})}. \tag{S6}$$

Similarly, for the 2nd dose vaccination,

$$f_{A2} = 1 - \frac{(1-e_{D2})(1-e_{S1})(1-f_{A1})}{(1-e_{S2})}. \tag{S7}$$

The fraction of deaths depends on the infection fatality ratio, which is related to the age of the infected individuals as follows (Bubar et al., 2021):

$$f_{SD} = \frac{IFR}{1-f_A}, \tag{S8}$$

where IFR is the infection fatality ratio rerating to the age as (Bubar et al., 2021; Levin et al., 2020):

$$\log_{10}(IFR) = -3.27 + (0.0524 \times Age) \tag{S9}$$

In our vaccination simulations, we initially assumed that all available vaccines would be administered as the first dose from the start of the vaccination campaign ($t = 0$) until the end of the specified time interval ($t = d_{\mathrm{dose}}$). Subsequently, any remaining vaccine supplies were allocated for the second dose. The vaccination process reverted to administering the first dose once there were no more individuals awaiting their second dose. This alternating pattern of first and second doses continued until the desired vaccination coverage was attained. The vaccine rollout rates for the first (α_{1i}) and second (α_{2i}) doses within age group i at each time step were computed as follows:

For the 1st dose period ($t = [xd_{dose}+1, (x+1)\, d_{dose}]$, x = 0, 2, 4, 6, …),

$$\alpha_{1i} = \frac{rollout}{\sum_{i=a}^{b}(S_i+E_i+A_i+R_{Ai})}, \tag{S10}$$

$$\alpha_{2i} = 0. \tag{S11}$$

For the 2nd dose period ($t = [xd_{dose}+1, (x+1)\, d_{dose}]$, x = 1, 3, 5, 7, …),

$$\alpha_{1i} = 0, \tag{S12}$$

$$\alpha_{2i} = \frac{rollout}{\sum_{i=a}^{b}(S_{p1i}+E_{p1i}+A_{p1i}+R_{Ap1i})}, \tag{S13}$$

where "*rollout*" represents the number of available vaccine doses each day. The parameters a and b correspond to the lower and upper age bands associated with different prioritization strategies: no prioritization for individuals aged 20 years and higher (a=5 and b=16), elder prioritization (a=13 and b=16), and worker prioritization (a=5 and b=*12*). Note that vaccines were not administered to individuals below 20 years of age (i = 1 - 4). The values of d_{dose} for the CV vaccine and AZ vaccine are 21 and 84 days, respectively, while the d_{dose} for the heterologous vaccine is 21 days.

C. COVID-19 vaccine doses administered per 100 people

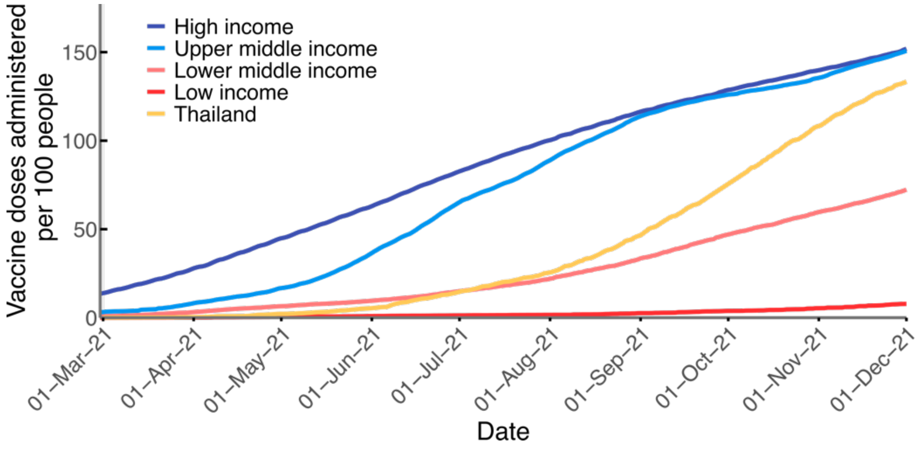

Figure S1. COVID-19 vaccine doses administered per 100 people by income groups. All doses, including boosters, are counted individually. As the same person may receive more than one dose, the number of doses can be higher than the number of people in the population (Mathieu et al., 2021).

D. Sensitivity analysis of the effect of the prioritization strategies on cumulative cases and deaths

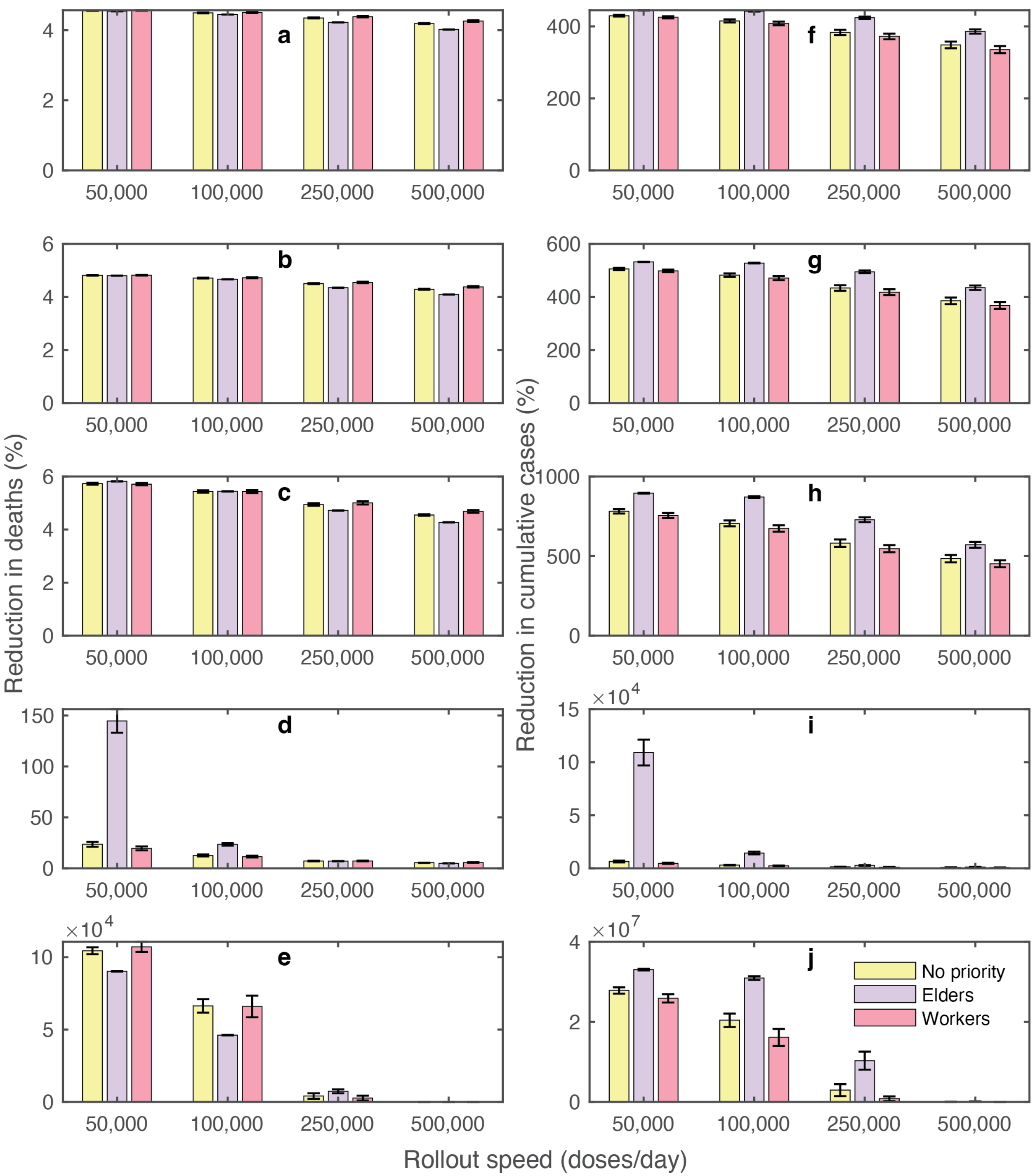

Figure S2. Effects of vaccine prioritization strategies on cumulative deaths and cases. Panels **(a-e)** display the cumulative death toll under different vaccine distribution speeds for scenarios

with *R* values of 1.05, 1.1, 1.2, 1.4, and 2.0, respectively. Panels **(f-j)** illustrate the cumulative case count for the same scenarios. Error bars represent variations in cumulative cases and deaths stemming from uncertainties in vaccine efficacy.

References

Bubar, K. M., Reinholt, K., Kissler, S. M., Lipsitch, M., Cobey, S., Grad, Y. H., & Larremore, D. B. (2021). Model-informed COVID-19 vaccine prioritization strategies by age and serostatus. *Science*, *371*(6532), 916-921. https://doi.org/10.1126/science.abe6959

Byambasuren, O., Cardona, M., Bell, K., Clark, J., McLaws, M.-L., & Glasziou, P. (2020). Estimating the extent of asymptomatic COVID-19 and its potential for community transmission: systematic review and meta-analysis. *medRxiv*, 2020.2005.2010.20097543. https://doi.org/10.1101/2020.05.10.20097543

Davies, N. G., Klepac, P., Liu, Y., Prem, K., Jit, M., group, C. C.-w., & Eggo, R. M. (2020). Age-dependent effects in the transmission and control of COVID-19 epidemics. *Nat Med*, *26*(8), 1205-1211. https://doi.org/10.1038/s41591-020-0962-9

Eyre, D. W., Taylor, D., Purver, M., Chapman, D., Fowler, T., Pouwels, K. B., Walker, A. S., & Peto, T. E. (2021). The impact of SARS-CoV-2 vaccination on Alpha & Delta variant transmission. *medRxiv*, 2021.2009.2028.21264260. https://doi.org/10.1101/2021.09.28.21264260

Eyre, D. W., Taylor, D., Purver, M., Chapman, D., Fowler, T., Pouwels, K. B., Walker, A. S., & Peto, T. E. (2022). Effect of Covid-19 vaccination on transmission of alpha and delta variants. *New England Journal of Medicine*, *386*(8), 744-756.

Fadlyana, E., Rusmil, K., Tarigan, R., Rahmadi, A. R., Prodjosoewojo, S., Sofiatin, Y., Khrisna, C. V., Sari, R. M., Setyaningsih, L., & Surachman, F. (2021). A phase III, observer-blind, randomized, placebo-controlled study of the efficacy, safety, and immunogenicity of SARS-CoV-2 inactivated vaccine in healthy adults aged 18–59 years: an interim analysis in Indonesia. *Vaccine*, *39*(44), 6520-6528.

Hitchings, M. D. T., Ranzani, O. T., Dorion, M., D'Agostini, T. L., de Paula, R. C., de Paula, O. F. P., de Moura Villela, E. F., Torres, M. S. S., de Oliveira, S. B., Schulz, W., Almiron, M., Said, R., de Oliveira, R. D., Silva, P. V., de Araújo, W. N., Gorinchteyn, J. C., Andrews, J. R., Cummings, D. A. T., Ko, A. I., & Croda, J. (2021). Effectiveness of ChAdOx1 vaccine in older adults during SARS-CoV-2 Gamma variant circulation in São Paulo. *Nature Communications*, *12*(1), 6220. https://doi.org/10.1038/s41467-021-26459-6

Jara, A., Undurraga, E. A., González, C., Paredes, F., Fontecilla, T., Jara, G., Pizarro, A., Acevedo, J., Leo, K., Leon, F., Sans, C., Leighton, P., Suárez, P., García-Escorza, H., & Araos, R. (2021). Effectiveness of an Inactivated SARS-CoV-2 Vaccine in Chile. *New England Journal of Medicine*, *385*(10), 875-884. https://doi.org/10.1056/NEJMoa2107715

Levin, A. T., Hanage, W. P., Owusu-Boaitey, N., Cochran, K. B., Walsh, S. P., & Meyerowitz-Katz, G. (2020). Assessing the age specificity of infection fatality rates for COVID-19: systematic review, meta-analysis, and public policy implications. *Eur J Epidemiol*, *35*(12), 1123-1138. https://doi.org/10.1007/s10654-020-00698-1

Lipsitch, M., & Kahn, R. (2021). Interpreting vaccine efficacy trial results for infection and transmission. *Vaccine*, *39*(30), 4082-4088.

Lopez Bernal, J., Andrews, N., Gower, C., Gallagher, E., Simmons, R., Thelwall, S., Stowe, J., Tessier, E., Groves, N., Dabrera, G., Myers, R., Campbell, C. N. J., Amirthalingam, G., Edmunds, M., Zambon, M., Brown, K. E., Hopkins, S., Chand, M., & Ramsay, M. (2021). Effectiveness of Covid-19 Vaccines against the B.1.617.2 (Delta) Variant. *New England Journal of Medicine*, *385*(7), 585-594. https://doi.org/10.1056/NEJMoa2108891

Mathieu, E., Ritchie, H., Ortiz-Ospina, E., Roser, M., Hasell, J., Appel, C., Giattino, C., & Rodés-Guirao, L. (2021). A global database of COVID-19 vaccinations. *Nature Human Behaviour*, *5*(7), 947-953. https://doi.org/10.1038/s41562-021-01122-8

Moghadas, S. M., Vilches, T. N., Zhang, K., Nourbakhsh, S., Sah, P., Fitzpatrick, M. C., & Galvani, A. P. (2021). Evaluation of COVID-19 vaccination strategies with a delayed second dose. *PLoS Biol*, *19*(4), e3001211. https://doi.org/10.1371/journal.pbio.3001211

Organization, W. H. (2021, Arpil). *Evidence Assessment: Sinovac/CoronaVac COVID-19 vaccine*. https://cdn.who.int/media/docs/default-source/immunization/sage/2021/april/5_sage29apr2021_critical-evidence_sinovac.pdf.

Palacios, R., Batista, A. P., Albuquerque, C. S. N., Patiño, E. G., Santos, J. d. P., Tilli Reis Pessoa Conde, M., Piorelli, R. d. O., Pereira Júnior, L. C., Raboni, S. M., & Ramos, F. (2021). Efficacy and safety of a COVID-19 inactivated vaccine in healthcare professionals in Brazil: the PROFISCOV study.

Pritchard, E., Matthews, P. C., Stoesser, N., Eyre, D. W., Gethings, O., Vihta, K.-D., Jones, J., House, T., VanSteenHouse, H., & Bell, I. (2021). Impact of vaccination on new SARS-CoV-2 infections in the United Kingdom. *Nature medicine*, *27*(8), 1370-1378.

Ssentongo, P., Ssentongo, A. E., Voleti, N., Groff, D., Sun, A., Ba, D. M., Nunez, J., Parent, L. J., Chinchilli, V. M., & Paules, C. I. (2022). SARS-CoV-2 vaccine effectiveness against infection, symptomatic and severe COVID-19: a systematic review and meta-analysis. *BMC infectious diseases*, *22*(1), 1-12.

Voysey, M., Clemens, S. A. C., Madhi, S. A., Weckx, L. Y., Folegatti, P. M., Aley, P. K., Angus, B., Baillie, V. L., Barnabas, S. L., Bhorat, Q. E., Bibi, S., Briner, C., Cicconi, P., Collins, A. M., Colin-Jones, R., Cutland, C. L., Darton, T. C., Dheda, K., Duncan, C. J. A., . . . Oxford, C. V. T. G. (2021). Safety and efficacy of the ChAdOx1 nCoV-19 vaccine (AZD1222) against SARS-CoV-2: an interim analysis of four randomised controlled trials in Brazil, South Africa, and the UK. *Lancet*, *397*(10269), 99-111. https://doi.org/10.1016/S0140-6736(20)32661-1
